# Genetic analysis of variation in lifespan using a multiparental advanced intercross *Drosophila* mapping population

**DOI:** 10.1186/s12863-016-0419-9

**Published:** 2016-08-02

**Authors:** Chad A. Highfill, G. Adam Reeves, Stuart J. Macdonald

**Affiliations:** 1 Department of Molecular Biosciences, University of Kansas, 1200 Sunnyside Avenue, Lawrence, KS 66045 USA; 2 Center for Computational Biology, University of Kansas, 2030 Becker Drive, Lawrence, KS 66047 USA

**Keywords:** Aging, Lifespan, QTL mapping, RNAseq, Complex traits, Multiparental populations

## Abstract

**Background:**

Considerable natural variation for lifespan exists within human and animal populations. Genetically dissecting this variation can elucidate the pathways and genes involved in aging, and help uncover the genetic mechanisms underlying risk for age-related diseases. Studying aging in model systems is attractive due to their relatively short lifespan, and the ability to carry out programmed crosses under environmentally-controlled conditions. Here we investigate the genetic architecture of lifespan using the *Drosophila* Synthetic Population Resource (DSPR), a multiparental advanced intercross mapping population.

**Results:**

We measured lifespan in females from 805 DSPR lines, mapping five QTL (Quantitative Trait Loci) that each contribute 4–5 % to among-line lifespan variation in the DSPR. Each of these QTL co-localizes with the position of at least one QTL mapped in 13 previous studies of lifespan variation in flies. However, given that these studies implicate >90 % of the genome in the control of lifespan, this level of overlap is unsurprising. DSPR QTL intervals harbor 11–155 protein-coding genes, and we used RNAseq on samples of young and old flies to help resolve pathways affecting lifespan, and identify potentially causative loci present within mapped QTL intervals. Broad age-related patterns of expression revealed by these data recapitulate results from previous work. For example, we see an increase in antimicrobial defense gene expression with age, and a decrease in expression of genes involved in the electron transport chain. Several genes within QTL intervals are highlighted by our RNAseq data, such as *Relish*, a critical immune response gene, that shows increased expression with age, and *UQCR-14*, a gene involved in mitochondrial electron transport, that has reduced expression in older flies.

**Conclusions:**

The five QTL we isolate collectively explain a considerable fraction of the genetic variation for female lifespan in the DSPR, and implicate modest numbers of genes. In several cases the candidate loci we highlight reside in biological pathways already implicated in the control of lifespan variation. Thus, our results provide further evidence that functional genetics tests targeting these genes will be fruitful, lead to the identification of natural sequence variants contributing to lifespan variation, and help uncover the mechanisms of aging.

**Electronic supplementary material:**

The online version of this article (doi:10.1186/s12863-016-0419-9) contains supplementary material, which is available to authorized users.

## Background

Life expectancy in developed countries has markedly increased in the last 100 years, and individuals born in the USA in 2011 can expect to live to nearly 80 years old [1]. Since old age is a major risk factor for an array of diseases [2], the prevalence of age-related disorders is concomitantly increasing as populations age. Given the significant segregating genetic variation for lifespan within populations [3], with twin studies indicating modest heritabilities of approximately 20–30 % [4, 5], a key challenge for biomedical science is to understand the genetic basis of variation in lifespan, and articulate any mechanistic relationships between aging and the risk for age-related disease.

To localize genes and/or variants associated with age in humans researchers have frequently used a GWAS (Genomewide Association Study) approach, comparing a cohort of centenarians to a cohort of middle-aged controls. Studies of this type have repeatedly associated age with variation at the *APOE* locus [6–8], a gene also known to strongly influence risk for Alzheimer’s [9]. However, such studies are often small due to the difficulty obtaining large cohorts of aged individuals, and thus lack power [10]. They also encounter the same problems as all GWAS, in that rare causative variants, and genes that segregate for a heterogeneous set of disease-causing alleles, are essentially invisible to the standard analytical methods employed [11–13]. In addition, direct genetic analysis of aging in humans must be carried out in the face of considerable environmental heterogeneity among samples.

One alternative fruitful strategy to discover the genetic and environmental determinants of variation in aging has been to use model systems, where total lifespan is much shorter than in humans, powerful genetic mapping experiments can be carried out using specifically bred individuals, in vivo genetic manipulation is possible, the environment throughout lifespan can be regulated to a large degree, and environmental interventions can be evaluated easily. Work in a number of non-human systems - from yeast, to flies, to mice - has demonstrated that dietary restriction routinely extends lifespan [14], and trials of dietary restriction in humans have yielded beneficial health responses [15, 16]. In addition, mutations in members of the insulin signaling pathway show robust effects on lifespan in several systems, such as *C. elegans* [17, 18], *Drosophila* [19], and mice [20]. Such observations suggest shared physiological mechanisms may underlie the response to aging, and imply some level of conservation in the genetic mechanisms contributing to lifespan variation.

In model systems, two broad strategies can be implemented to identify genes and pathways impacting lifespan and age-related phenotypes: Mutational analyses, and mapping loci contributing to variation in lifespan in natural, or semi-natural laboratory populations. Given the relative ease with which large-effect mutations can be generated and interrogated in flies, multiple studies have screened large sets of induced mutations for their effects on lifespan (e.g., [21, 22]), and detailed mechanistic studies targeting specific genes and pathways have added considerably to our understanding of the aging process. However, such loci may be distinct from those that harbor naturally-segregating sites underlying variation in lifespan (compare Tables one, two, and three in [23]). To identify genes contributing to natural variation in lifespan, *Drosophila* researchers have used techniques such as QTL (Quantitative Trait Locus) mapping [24] to screen the genome in an unbiased fashion, and - coupled with downstream functional tests - have successfully implicated a small number of genes in the control of lifespan variation (e.g., *Dopa decarboxylase*, [25]).

A concern with many previous QTL mapping studies is that they employ mapping populations initiated with just two strains, and use individuals subjected to very few rounds of meiotic recombination, limiting the scope of the genetic variation interrogated, and limiting the mapping resolution achievable (e.g., [26]). Here, we employ the DSPR (*Drosophila* Synthetic Population Resource [27, 28]) - a multiparental, advanced intercross panel of RILs (Recombinant Inbred Lines) - to dissect genetic variation in lifespan in mated female *Drosophila*, resolving five modest-effect QTL to relatively short genomic regions (0.1–1.2Mb). We also use RNAseq to identify genes showing differential expression between young and old animals in a subset of DSPR lines. The set of genes exhibiting age-related changes in gene expression in our study shows significant overlap with previous such studies in flies, and implicates a small number of highly plausible aging candidate genes within mapped QTL. Some of the loci we highlight were already considered candidates to contribute to aging based on studies of induced mutations, for instance *Relish*, a gene known to be involved in immune response.

## Methods

### Mapping population

The DSPR is a large panel of RILs derived from a multi-parental, advanced generation intercross [28]. Each of the two populations - pA and pB - was initiated from a set of eight, highly-inbred founders, and was maintained as a pair of independent subpopulations - pA1, pA2, pB1, and pB2 - for 50 generations. Subsequently ~800 RILs per population were established via 25 generations of full sib mating, and genotyped via Restriction site Associated DNA sequencing (RADseq). Since all founder lines were also sequenced to 50X coverage, we were able to use a hidden Markov Model (HMM) to elucidate the mosaic founder structure of each RIL. Full details of the construction of the DSPR are presented in King et al. [28].

### Lifespan assay

Briefly, our assay was conducted as follows: Each RIL was copied from our stock collection in a single vial, and in the next generation expanded to two replicate experimental vials. Nine days after egg laying any emerged adults were cleared from experimental vials. After 48 h, 0–2 day old flies were transferred to fresh media, and held for 24 h to ensure mating. Subsequently, 30 mated 1–3 day old female flies per RIL were collected under CO_2_ anesthesia into a single assay vial. Flies were transferred to fresh media every two days for the first two weeks of life, and every three days thereafter, and flies were scored daily until half the females were dead. We tracked vials and genotypes using systems of anonymous barcodes, a barcode reader, and custom R code (r-project.org) designed to record the number of dead flies each day, trace all anonymous barcodes back to the original RIL genotype, and find the median lifespan for females from each RIL assayed.

We collected median lifespan data for mated females from 805 pB DSPR RILs, testing each RIL in one of four experimental blocks (150–233 RILs per block; Additional file 1: Table S1). To minimize technical and environmental variation across blocks we ensured that adults were cleared from experimental vials to maintain similar egg density across vials, maintained the exact same experimental timing (as described above) for each block, and conducted all fly rearing and maintenance on a 12 h light/12 h dark cycle at 25 °C and 50 % relative humidity, using cornmeal-molasses-yeast media in standard, narrow *Drosophila* vials.

### QTL mapping

The analytical framework used to identify QTL in the DSPR is described in detail in King et al. [28], and the power and properties of the mapping approach is presented in King et al. [27]. Briefly, the HMM assigns to each region in each RIL a probability the genotype is one of 36 possible homo- or heterozygous states. Since the vast majority of the positions in the RILs are homozygous, we generate eight additive homozygous probabilities per position, and regress RIL median lifespan on these probabilities. Since we see variation among experimental blocks (Additional file 2: Figure S1) we additionally include “block” as a covariate. We note that because lines from the pB1 and pB2 subpopulations were segregated into different blocks for the lifespan assay, some of the block-to-block variation is likely due to differences between subpopulations in addition to technical, experimental variation.

QTL were identified as peaks reaching a 5 % genomewide, permutation-derived threshold [29], and we used 2-LOD support intervals to put confidence intervals on the true positions of QTL [27]. All mapping was carried out using the DSPRqtl R package (github.com/egking/DSPRqtl; FlyRILs.org).

### RNAseq

In the course of assaying lifespan we collected samples of young (1–3 days old) and old (median lifespan for genotype) females from a fraction of the RILs. Each experimental sample consisted of a group of 10 females of the same genotype collected under CO_2_ anesthesia and snap-frozen using liquid nitrogen. For each sample to be used for RNAseq we removed heads from bodies (thorax + abdomen) by vortexing tubes containing frozen female flies, separating heads and bodies with a paintbrush over a dry ice-cooled aluminum block. RNA was isolated from each tissue sample using TRIzol reagent (15596-018, ThermoFisher Scientific) following the manufacturers protocol, except that for head samples we scaled down all volumes to 1/4 of the recommended amounts.

To examine expression in bodies we selected 10 RILs with a relatively short lifespan, and 10 with longer lifespan (Additional file 3). Equal amounts of total RNA from each of the appropriate 10 samples were combined to generate four pools; short-lived/young, short-lived/old, long-lived/young, and long-lived/old. Each pool was then cleaned through an RNeasy Mini column (74104, Qiagen), used to generate a standard TruSeq RNAseq library (version 2, Illumina), and sequenced on an Illumina HiSeq 2500 instrument (KU Genome Sequencing Core) to generate single-end 100bp reads (see SRA accession SRP072382). Quality trimming via sickle (version 1.200, github.com/najoshi/sickle) resulted in 34.2–39.5 million reads per sample. We used TopHat (version 2.0.12, tophat.cbcb.umd.edu; [30, 31]) to assemble reads to the *D. melanogaster* reference genome (NCBI build 5.3, tophat.cbcb.umd.edu/igenomes.shtml), resulting in 84.0–87.1 % reads aligning, and Cuffdiff (version 2.1.1, cufflinks.cbcb.umd.edu; [32–34]) to identify differentially expressed genes in four pairwise contrasts (short-lived/young *versus* short-lived/old, long-lived/young *versus* long-lived/old, short-lived/young *versus* long-lived/young, and short-lived/old *versus* long-lived/old). We consider a gene to be differentially expressed if it survives a genomewide, per contrast Benjamini-Hochberg 5 % False Discovery Rate (FDR) correction for multiple testing.

To investigate expression in heads we selected six genotypes (Additional file 3), made RNAseq libraries for the six pairs of young and old head samples, and sequenced to generate paired-end 50bp reads (see SRA accession SRP072396). Following quality trimming we had 14.1–26.0 million read pairs per sample, and genome alignment resulted in 78.8–90.9 % reads mapping. Statistical testing was carried out to find genes differentially expressed (FDR = 5 %) between the heads of young and old flies, treating the separate RIL genotypes as replicates.

## Results

### Variation in lifespan in the DSPR

**Fig. 1:**
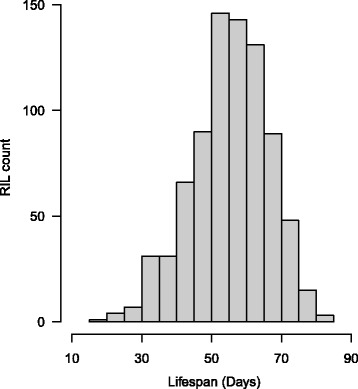
Distribution of female lifespan among DSPR RILs. We assayed lifespan for 805 RILs from the DSPR, measuring the phenotype as the time required for half the flies to die

We observed substantial lifespan variation among the 805 DSPR RILs tested (Fig. 1, Additional file 1: Table S1), with median mated female lifespan averaging 55.0 days, ranging from 16.4–80.6 days across RILs. Since each RIL was assayed in just one block, some fraction of this variation is due to environmental and technical variation across blocks, such as uncontrolled micro-environmental variation across assay vials (Additional file 2: Figure S1). Nonetheless, the scale of lifespan variation we see is remarkably similar to that observed in a screen of virgin females from 197 *Drosophila* Genetic Reference Panel, DGRP lines (mean = 55.3 days, range = 22.1–80.3 days; [35]).

Given the number of RILs tested, to streamline phenotype data collection we elected to score RILs for median lifespan, allowing us to discard assay vials at that point, and avoid waiting for all flies in a vial to die. Although our data collection pipeline did not allow the calculation of mean lifespan for each RIL, results from the DGRP show that the correlation between mean and median lifespan for a set of inbred lines is very strong (*r* = 0.97, *p* < 10^−15^; [35]). One caveat with our use of a phenotype based on the median lifespan from a single replicate vial per genotype is that we are unable to estimate heritability for lifespan in the DSPR.

### QTL for variation in lifespan

**Fig. 2:**
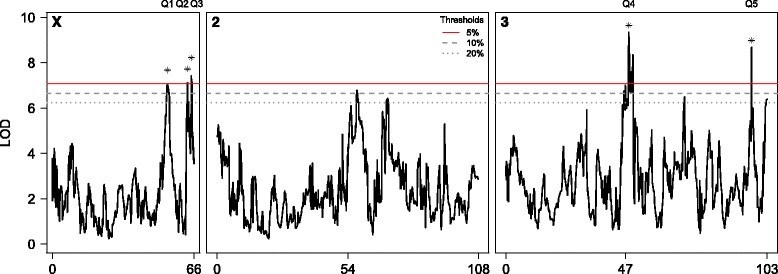
Genome scan for lifespan QTL. The black solid line indicates the LOD score following a scan for QTL contributing to variation in lifespan in the DSPR. The *x*-axis indicates genetic distance, and genetic positions 54 and 47 are the sites of the centromeres on chromosomes 2 and 3, respectively. The red line is a permutation-based genomewide 5 % threshold (LOD = 7.08). Five QTL show peaks with LOD scores higher than this threshold, their positions are indicated with asterisks, and the codes Q1-Q5 used throughout the text are provided above the plot. We also provide genomewide 10 % (gray dashed line, LOD = 6.64) and 20 % (gray dotted line, LOD = 6.24) thresholds. Peaks surviving these more liberal thresholds (at 57cM and 70cM on 2R, and 70cM and 103cM on 3R) are less compelling candidates to contribute to lifespan variation

We mapped five QTL for lifespan in the DSPR (Fig. 2, Table 1, Additional file 4: Table S2) that survive a 5 % permutation-derived statistical threshold. Each QTL explains a modest fraction of the among-line variation for lifespan (4.0–5.2 %, Table 1), and assuming the QTL are independent and act additively, collectively explain 22.2 % of the genetic variation for lifespan in the DSPR. With 800 RILs the power to identify common biallelic or multiallelic QTL contributing 5 % to the total variation in the RIL panel is 80–90 % [27]. This implies that any undetected genetic factors contributing to lifespan variation in the DSPR either have small effects on variation, or are rare in the panel. A number of LOD peaks do survive more liberal genomewide thresholds (Fig. 2, Additional file 4: Table S2), and could represent such factors, although our confidence in these peaks is limited, and we do not consider them further.

**Table 1:** Lifespan QTL mapped in the pB DSPR panel

QTL	Peak LOD ^*a*^	Chr. ^*b*^	Physical interval (Mb) ^*b*^	Cytological interval ^*b*^	Number of genes ^*c*^	Variation explained ^*d*^
Q1	7.1	X	16.0–16.7	14A6–15A3	84	4.0
Q2	7.1	X	19.5–20.2	18C8–19C1	93	4.0
Q3	7.4	X	20.9–21.4	19E4–20A1	51	4.2
Q4	9.4	3R	8.1–9.3	84F1–85D11	155	5.2
Q5	8.7	3R	28.7–28.8	98E2–98E5	11	4.9

^*a*^ LOD score at the QTL peak

^*b*^ The chromosome arm on which the QTL resides, the physical position of the QTL interval (defined as a 2-LOD drop from the peak) in the *D. melanogaster* reference genome release 6, and the equivalent cytological interval

^*c*^ Number of protein-coding genes present within the QTL interval

^*d*^ The fraction of the among-line variation explained by the QTL

**Fig. 3:**
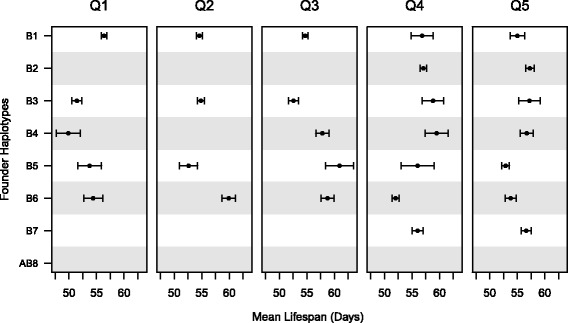
Founder allele strain effects at mapped lifespan QTL. Phenotype means (±1 standard error) are presented for each founder at each QTL peak. Data is presented only for those founders present in at least 10 RILs at a probability > 0.95

A feature of multi-parental mapping panels such as the DSPR is that we can estimate the effects of each founder allele at mapped QTL, and can determine those founders that are likely to harbor alleles contributing to long lifespan. Figure 3 shows the founder allele effects for all five mapped QTL. It is not obvious from this plot that loci contributing to lifespan variation generally segregate for two alleles (e.g., a “high” and a “low” allele), and instead may segregate for multiple alleles, each with different effects on phenotype. Of course, since our QTL are mapped to intervals containing multiple genes (Table 1) we cannot discount the possibility that mapped QTL are due to the action of multiple genes. Regardless, it is possible to identify pairs of founders that appear to harbor haplotypes with contrasting effects on lifespan. For example, RILs carrying genetic material from founders B5 and B6 at Q2 have relatively low, and relatively high lifespan, respectively (Fig. 3). Genetic differences between these founders in the Q2 interval are likely to be enriched for variants causally contributing to lifespan.

The five QTL are mapped to regions encompassing 660kb (Q1), 660kb (Q2), 510kb (Q3), 1.2Mb (Q4), and 80kb (Q5) of the *D. melanogaster* genome (Table 1). The Q4 interval is relatively large since this QTL resides near the chromosome 3 centromere where recombination is suppressed. Aside from Q4, QTL intervals include 11–93 protein-coding genes (Table 1, Additional file 5: Table S3). To determine whether any of the genes encompassed by mapped QTL have previously been implicated in aging and/or lifespan regulation, we searched FlyBase [36] to identify genes tagged with controlled vocabularies that included the words “aging”, “lifespan”, “lived”, and “longevity” (Additional file 6). We identified a total of 568 candidate genes (Additional file 7: Table S4), 14 of which reside within QTL intervals (Table 2).

**Table 2:** FlyBase aging candidate genes within mapped QTL

QTL ^*a*^	Gene name	Symbol
Q1 ^*b*^	*cabeza*	*caz*
	*hangover*	*hang*
	*methuselah-like 1*	*mthl1*
Q2	*carnation*	*car*
	*CG18809*	–
	*domeless*	*dome* ^*c*^
	*Ubiquilin*	*Ubqn* ^*c*^
	*Zwischenferment*	*Zw* ^*c*^
Q3	*Cystathionine beta-synthase*	*Cbs*
	*Microsomal glutathione S-transferase-like*	*Mgstl*
Q4	*Coenzyme Q biosynthesis protein 2*	*Coq2*
	*Insulator binding factor 2*	*Ibf2*
	*pumilio*	*pum*
	*Relish*	*Rel* ^*c*^

^*a*^ No genes from our FlyBase controlled vocabulary searches were present within the Q5 interval

^*b*^ The gene *CG32576*, which resides within Q1, was also tagged in our FlyBase search as “short lived” but this appears to be an annotation error [66]

^*c*^ These genes were also shown to increase in expression with age in female heads in our RNAseq study

### Comparison with previous mapping studies

Candidate aging genes extracted from FlyBase are often associated with longevity based on mutant phenotypes (e.g., *Cbs*, [37]), and may or may not harbor naturally-segregating variation affecting lifespan. Thus, we sought to compare our data to previous studies mapping lifespan loci among naturally-derived chromosomes. A number of previous studies have used various mapping designs to identify QTL contributing to variation in lifespan and aging in *D. melanogaster* [26, 38–49], and all five of the QTL we map in the DSPR overlap with at least one QTL mapped in a prior study (Additional file 8: Figure S2). While this observation gives some additional confidence in our phenotype and mapping, we note that the 13 studies we highlight mapped well over 100 QTL, and mapped intervals that collectively implicate 93.4 % of the *D. melanogaster* genome (Additional file 8: Figure S2). This phenomenon of aging QTL implicating large fractions of the *Drosophila* genome has been noted previously [39]. Using a resampling procedure we tested how often five non-overlapping, randomly-positioned QTL of the same physical size as the set mapped in this study overlapped previously identified QTL; Over 1000 runs, 85 % of the time each of the five simulated QTL overlap at least one QTL mapped in a prior study, implying the overlap we see in our real data is expected.

The complexity of the genetic architecture of the phenotype may go some way to explaining the observation that mapped aging QTL blanket the genome. Lack of resolution in QTL mapping studies using animals that have passed through a small number of generations of meiotic recombination is also likely an important factor determining the large fraction of the genome that lifespan QTL mapping studies collectively implicate. In addition, differences in the biology of the aging traits under study certainly contributes to the differences in the QTL identified; There are clear sex differences is the genetic control of many traits, including lifespan [26], mating status affects lifespan [46], and there is ample evidence of genotype-by-environment interaction underlying variation in lifespan [43, 47].

A more high-resolution study was conducted by Burke et al. [50]. Using animals from the highly-recombinant “synthetic” 8-way populations from which the DSPR was derived, they compared allele frequencies in extremely old cohorts of females to those from randomly-selected, control females. Across all replicate populations Burke et al. [50] identified eight regions surviving a 5 % false positive rate, but none of these overlap with the QTL we map here. Overlap remains very limited even when considering an additional eight regions identified by Burke et al. [50] that only survive a very liberal 50 % false positive rate threshold in their study; Just one such region overlaps with our Q4 at the chromosome 3 centromere.

Ivanov et al. [35] recently used the DGRP to carry out a genomewide association study for lifespan using virgin females. Although no variant in the SNP-based GWAS, and no gene in the gene-based GWAS, survived a correction for multiple testing, likely due to the low power of the DGRP design [51], a number of variants and genes showed nominally-significant association tests at *P* < 10^−5^. Such tests may be enriched for true causative variants/genes. Of the 50 SNP association tests with the lowest *P*-values, just one is within a region implicated by a QTL mapped in this study, a variant present within the *bves* gene [35] that is within our Q3. Although there is no specific information regarding the effect of *bves* on lifespan in FlyBase [36], an insertion mutation in the gene has been shown to increase the susceptibility of *Drosophila* to the fungal pathogen, *Metarhizium anisopliae* [52]. None of the top-ranked gene-based DGRP burden-type tests carried out by Ivanov et al. [35] fall within our QTL intervals.

### Regulatory candidate genes for lifespan

It is likely that some fraction of the sites that contribute to among-individual variation in a complex phenotype are regulatory in origin [53, 54]. Thus, we employed two RNAseq studies; The first experiment was designed to identify genes differentially expressed in body tissue between young and old female flies, and to additionally find differential expression between long-lived and short-lived genotypes. The second experiment was tailored to identify genes differentially expressed between the heads of young and old animals. Any candidate genes we identify may plausibly harbor functional regulatory variants impacting lifespan.

For the body RNAseq we extracted RNA from samples of young and old flies from ten long-lived and ten short-lived RILs, mixed RNA to generate four pools each containing material from ten samples, generated and sequenced four libraries, and tested for differential gene expression in four pairwise contrasts: short-lived/young *versus* short-lived/old, long-lived/young *versus* long-lived/old, short-lived/young *versus* long-lived/young, and short-lived/old *versus* long-lived/old. After analysis we identified 155 genes differentially expressed between young and old flies in short-lived genotypes (22 down with age, 133 up with age), and 160 differentially expressed between young and old flies in long-lived genotypes (83 down with age, 77 up with age). Sixty-six genes overlap between these two sets, and all 66 show the same direction of age-related expression change in short- and long-lived animals, implying consistency in the pattern of age-related gene expression change across genotypes. We additionally identified 9 (16) genes showing significantly different expression in young (old) females when comparing short- and long-lived genotypes (Additional file 9: Table S5). Overall, 252 genes survive a genomewide FDR threshold of 5 % in at least one contrast.

For the head-specific RNAseq we extracted RNA from samples of young and old flies from six RILs, generated and sequenced separate libraries for each of the 12 samples, and identified 1,940 genes differentially expressed between young and old flies in heads (995 down with age, 945 up with age; Additional file 10: Table S6). Given that separate RILs were treated as replicates in the head RNAseq analysis, and assuming some consistency in the age-related patterns of expression across RILs, our power to detect small changes in expression in this head analysis is likely higher than for the body analysis that lacks replication at this level. Nonetheless, there was significant overlap - 130 genes - between the set of 249 genes showing differential expression between young and old flies in bodies, and the set of 1,940 showing expression differences between young and old flies in heads (Fisher’s Exact Test, *p* < 10^−15^, assuming 14,000 genes in the *D. melanogaster* genome). Nearly all - 128/130 - of the genes in this overlapping set show expression changes in the same direction in bodies and heads (genes *Mur2B* and *CG4377* show opposing age-related changes). Thus, despite experimental and analytical differences, we find similarity in the age-related patterns of expression across tissues.

Employing the Gene Ontology, GO (geneontology.org; [55, 56]) we classified genes showing differential expression by function and their involvement in particular biological processes (see Additional file 11 for a summary). In both the head and body datasets considered separately we found a significant enrichment of genes involved in defense and response to bacteria, recapitulating previous results [57]. We additionally found an enrichment of genes involved in egg coat formation in the body data only, finding 5/14 such genes, all of which decrease in expression with age (see also [57]). This is presumably associated with reduced reproductive output in older mated females, or a reduced capacity for egg production due to reproductive aging, since Lai et al. [58] observed a reduction in expression at three vitelline membrane genes (*Vm26Aa*, *Vm26Ab*, and *Vm34Ca*) in older virgin females. Finally, in bodies we saw an enrichment of myofibril assembly genes (10/40 genes found, all of which decrease in expression with age), and in heads an enrichment of genes involved in the electron transport chain (42/86 found, and 39/42 go down with age), both observations potentially reflecting a general loss of vigor with age. Studies in both mice and humans have also shown that many components of the electron transport chain show reduced expression with age [59].

Several other groups have previously used array-based expression profiling to identify genes that change with age in various *D. melanogaster* populations. We sought to compare the results of our study with this other work, and determine the extent of overlap in the genes identified among experiments. We extracted information on genes showing age-related changes in expression from Pletcher et al. [57], Landis et al. [60], Lai et al. [58], Zhan et al. [61], and Carlson et al. [62], converted all gene names to the most current FlyBase gene IDs (see Additional file 12), and examined for the number of overlapping genes. Overall, 83 % of the genes we identify as differentially expressed in bodies were identified in at least one other study, and 59 % of the genes we identify in heads replicated (Additional file 13: Figure S3). We assessed the statistical significance of overlap in the sets of genes identified using the R software package *SuperExactTest* [63] that can calculate the probability of intersection among any number of gene sets. Considering our head (252 genes) and body (1,940 genes) datasets separately, and assuming 14,000 total genes in the *Drosophila* genome, the number of genes that intersect between our study and three or more other sets of age-related genes is highly significant (all *p*-values < 3.7 × 10^−15^). Thus, while there are an array of biological and technical differences among studies, a core set of genes appear to be consistently identified as showing age-related changes in gene expression.

Pletcher et al. [57] found no evidence for any association between the chromosomal location of a gene and whether it exhibited an age-related change in expression. Our dataset exhibited a similar pattern, with differentially-expressed genes scattered throughout the genome (Additional file 14). Of considerable interest is whether any of the genes we identify in our RNAseq screen are present within genomic intervals implicated by mapped QTL. A total of 55/2,061 unique RNAseq candidates are present within these intervals; Two were identified only in our body experiment, 52 only in our head experiment, and one was observed in both studies (Additional file 15: Table S7). In all cases these genes were identified as differentially expressed with age, and none were found to be differentially expressed between short- and long-lived genotypes. Thirty-one of the 55 genes have been shown to have age-related changes in expression in previous studies, and 4/55 represent aging candidate genes identified in FlyBase (Table 2); *dome*, *Ubqn*, and *Zw* (all under Q2) and *Rel* (under Q4), all of which show increased expression in the heads of older females. Although differentially expressed genes are not enriched within QTL intervals - QTL collectively cover 2.5 % of the physical genome and harbor 2.7 % of the differentially-expressed genes we identified - under the assumption that loci impact lifespan variation via changes in expression, these handful of genes represent excellent candidates to harbor regulatory variation affecting lifespan.

## Discussion

We carried out an unbiased screen to identify loci segregating for allelic variation influencing lifespan of mated female *D. melanogaster*. By virtue of employing a multiparental advanced intercross population we were able to map putative aging genes to relatively small regions of the genome averaging 640kb (Table 1), aiding future resolution of the actual causative loci. We uncovered three X-linked and two autosomal QTL that collectively explain 22.3 % of the among-genotype variation in lifespan in the DSPR (Table 1). We were unable to estimate the heritability for lifespan directly in the DSPR, since our measure of lifespan is the median time of death of a single cohort of 30 flies from each RIL. Nonetheless, a previous estimate of the broad-sense heritability of lifespan in *Drosophila* is 0.41 [35], suggesting that the QTL we identify likely explain very small fractions of the total phenotypic variation for lifespan.

We followed up our QTL mapping with a pair of RNAseq screens, separately focusing on head and body tissue, to both examine changes in the regulatory landscape during aging, and resolve plausible candidate aging loci within mapped QTL. We identified a large array of genes with age-related changes in gene expression, observed significant overlap over tissues in the sets of genes identified, and many of the genes we identified had been previously found in other genomewide expression studies of lifespan [57, 58, 60–62]. Examination of the functions and molecular properties of the genes we identified revealed several broad patterns. Mostly notably we recapitulated the observation that antimicrobial genes increase in expression with aging in flies [57]. This likely reflects the observed increase in bacterial load in aged flies [64, 65], a phenomenon that may be directly associated with aging and mortality. We additionally found an enrichment of genes with functions in the electron transport chain, with such genes nearly always showing a reduction in gene expression in aged heads (Additional file 11). Zahn et al. [59] have argued that, as one of the only pathways identified to be age-related in humans, mice, and flies, reduction in expression of the electron transport chain components represents a common signature of aging.

### Resolving candidates contributing to natural variation in aging

A benefit of mapping with high resolution in an advanced intercross population is that modest numbers of genes are implicated, allowing plausible candidates to be highlighted for future experimental tests. Below we summarize those plausibly functional loci residing within each of our mapped QTL.

Q1 (14A6-15A3) overlaps with lifespan QTL identified in studies by Reiwitch & Nuzhdin [46] and Defays et al. [39], and several of the 84 genes implicated by Q1 have been previously implicated in aging in flies (Table 2). A *caz* deletion mutation exhibited reduced longevity in comparison to wildtype [66], as did a *hang* P-element insertion mutation [67]. In addition, copy number at the *meiotic 41* gene has been shown to affect lifespan [68]. The gene *methuselah-like 1* (*mthl1*) is annotated in FlyBase as being involved in the determination of adult lifespan [36], although this appears to be entirely due to the sequence similarity of this gene to *methuselah*, a classic aging candidate gene [69]. We also identified 14 genes that change in expression between young and old flies in the head (Additional file 15: Table S7). Notably *UQCR-14*, which appears to be involved in mitochondrial electron transport [70], shows decreased expression with age in our study, reduced expression with age in whole females in both regular food and caloric restriction conditions in Pletcher et al. [57], lower expression with age in whole males [60], and changes expression with age in brain-tissue derived from males [61].

Q2 (18C8-19C1) was found in the same position as QTL mapped in three previous studies [39, 46, 47], although the QTL we map is considerably smaller in size, implicating 93 protein-coding genes. Several strong aging candidate genes are present in this interval (Table 2). A point mutation in *car* shows significantly reduced lifespan in males [71], RNAi knockdown of the mitochondrial electron transport chain complex IV component gene *CG18809* leads to a 16–19 % increase in lifespan in female flies [72], a dominant negative version of *dome* increases mortality in a *G9a* mutant background [73], silencing *Ubqn* in the nervous system shortens lifespan in males and leads to neurodegeneration [74], and overexpression of *Zw* (glucose-6-phosphate dehydrogenase) increases lifespan [75]. *dome*, *Ubqn*, and *Zw* are also among the genes we identified as differentially expressed in heads between young and old animals, and these three genes all show enhanced expression with age (Additional file 15: Table S7).

Q3 (19E4-20A1) resides close to Q2 (Fig. 2), however the 2-LOD drop confidence intervals of the peaks do not overlap (Table 1), and the founder allele effect plots show different patterns (Fig. 3), so we can be reasonably confident the QTL represent separate loci. The positions of our Q1, Q2, and Q3 all overlap one of the broad QTL mapped by Defays et al. [39], highlighting the resolution of our study. Two *a priori* aging candidate genes are present within the Q3 interval (Table 2); *Cbs* overexpression leads to increased lifespan [37], and *Mgstl* null mutants exhibit reduced lifespan compared to wildtype controls [76].

Q4 (84F1-85D11) is the broadest peak we map, implicating 155 genes, likely because the QTL resides close to the chromosome 3 centromere, a site of reduced crossover rate. Our QTL overlaps loci previously mapped in five studies [39, 40, 43, 47, 49], although the region we implicate is substantially smaller than in most of these studies. Several genes in the Q4 interval have been previously implicated in *Drosophila* longevity (Table 2). *Coq2* is involved in the synthesis of Coenzyme Q (ubiquinone; [77]), an essential electron carrier in the mitochondrial electron transport chain. Heterozygous genotypes with just one functional copy of *Coq2* show lifespan extension in both males and females [77]. Genotypes with nonfunctional *Ibf2* are short-lived [78], there is some evidence for a slight reduction in lifespan in genotypes carrying a mutant for *pum* [79], and loss of function mutations in *Rel* - a gene critical in the induction of the immune response in flies - dramatically reduce survival time compared to controls [80]. *Rel* is also an excellent expression candidate for a role in lifespan regulation, since we found it to be increased in expression with age in heads (Additional file 15: Table S7), and three previous studies also showed increased *Rel* expression in older flies [57, 58, 60]. Q4 also harbors *polychaetoid*, the only gene identified in a P-element screen for lifespan extension mutations that overlapped our five QTL intervals [21]. A number of genes within Q4 show expression variation between young and old animals in our study (Additional file 15: Table S7). This set includes *CG8032*, which is also the only member of a set of 39 lifespan-reducing loci identified in a gain-of-function screen that is implicated by QTL mapped in the present study [22], and *Nmdmc*, overexpression of which has been shown to extend lifespan in flies [81]. Given the number of genes within Q4, and the ample evidence of multiple candidates present in the region, it is not unlikely that more than one gene in the region is responsible for the QTL we map.

Finally, we mapped Q5 (98E2-98E5) to a small interval on chromosome 3R containing just 11 genes (Table 1, Fig. 2). This region has previously been implicated in the control of lifespan [44–46], although no strong *a priori* candidates are present. One of the loci within the Q5 interval, *wdn*, shows an age-related increase in expression in heads in our study (Additional file 15: Table S7), although this result was not recapitulated in any of the five other expression datasets we examined.

### Replication among studies mapping naturally-segregating aging variants

Each of the five QTL we isolated in the DSPR co-localizes with the positions of QTL mapped for lifespan in at least one of the 13 other studies we examined (Additional file 8: Figure S2). It is clear from examining overlap among all studies that there is some commonality in the genomic regions implicated in the control of natural variation in aging. However, it is equally clear based on the lack of the overlap among studies with the highest level of resolution (this study along with [42, 45, 49]) that there are significant differences in the sets of loci implicated in different works (Additional file 8: Figure S2). Studies routinely employ different starting sets of genotypes, so at least some of the differences observed must be due to different mapping panels segregating for different subsets of functional allelic variation. However, differences in power among studies are also likely to play an important role in the differing results. It is most likely that aging is a highly polygenic trait, and that individual variants each underlie only a tiny fraction of lifespan variation, as evidenced by the small effects of the two genes replicated in multiple human GWAS for aging, *APOE* and *FOXO3A* [82]. If variant effects are routinely this low, even studies with reasonable sample sizes are likely to be underpowered; For instance, this study used 805 RILs, and has ~30 % power to identify QTL contributing 2.5 % to among-line variation in phenotype [27]. Thus, if the genetic architecture of lifespan is constructed from the effects of many, very small-effect variants, any given genomewide study may only find a small subset of the loci segregating for age-related variation.

A further important difference among studies in *Drosophila* is that the assays used to measure lifespan are frequently different, both at gross levels (e.g., studies may focus on different sexes), and at more subtle levels, such as any number of technical differences in the laboratory environments used to rear flies and maintain aging populations (e.g., temperature, media composition, larval density). Loci contributing to aging have been shown to be sex-specific in many cases [26, 44], whether the flies are mated or virgin can alter the QTL identified [46], and many QTL have been shown to be highly environment-dependent [43, 47]. If the effects of functional alleles at aging genes are typically, or even often sensitive to the environment in this way, i.e., exhibit genotype by environment interaction [83–85], we should expect to routinely identify different sets of variants in different studies, with variants only being identified under those conditions under which they have detectable effects on phenotype. A key benefit of a consistent, chemically-defined diet for flies [86] would be to help minimize lab-to-lab variation in studies of life history traits, help enhance replicability of genetic effects across studies, and promote understanding of the mechanisms by which allelic variation leads to variation in aging under a single set of conditions.

## Conclusions

Regardless of the precise set of aging loci identified in mapping populations of *Drosophila*, there is clearly consistency across studies in the pathways implicated in the aging process. This is most easily seen in the various expression profiling experiments that have been carried out, where core groups of genes robustly and consistently show age-related changes in expression, notably antimicrobial defense response genes that are routinely upregulated during aging, and genes involved in the electron transport chain that are routinely downregulated with age. Thus, there is hope that the genes implicated by QTL mapping studies, regardless of their differences across studies, could provide valuable inroads into a mechanistic understanding of the pathways involved in aging. In this regard, our identification of *UQCR-14*, a gene within Q1 that is involved in electron transport and shows a decrease in expression with age, *CG18809*, a gene within Q2 that encodes a component of the electron transport chain, and *Relish*, a gene under Q4 that is involved in mobilizing the antimicrobial response, and shows increased expression in aged animals, represent excellent candidates for future functional analysis, and to identify causative sequence-level variation underlying aging. The prospects for direct functional validation of age-related variation in model systems via allele swapping - moving “high” alleles into “low” backgrounds and *vice versa* - using CRISPR-Cas9 editing are strong, and will obviate the need to “validate” natural allelic effects with synthetic constructs (e.g., RNAi). The ability to examine whole organism phenotypes, in addition to cellular and physiological phenomena, in specifically edited animal models is a considerable strength of model organisms that will allow the exploration of aging pathways that may also be implicated in humans.

## Abbreviations

DGRP, *Drosophila* genetic reference panel; DSPR, *Drosophila* synthetic population resource; FDR, false discovery rate; GO, gene ontology, GO; GWAS, genomewide association study; HMM, hidden Markov model; LOD, logarithm of odds; QTL, quantitative trait locus; RADseq, restriction site associated DNA sequencing; RILs, recombinant inbred lines; RNAseq, RNA sequencing.

## Additional files

Additional file 1: Table S1. Median lifespan measurements for all DSPR RILs tested. The stock code for each line is provided in the “RIL” column, the median lifespan for mated females in hours is presented in “MedLifespanHrs”, and the “Block” column represents the experimental block (1–4) the line was assayed in. (TXT 15 kb) Additional file 2: Figure S1. Block-to-block variation in lifespan. (PDF 43 kb) Additional file 3: RILs selected for RNAseq analysis. (PDF 28 kb) Additional file 4: Table S2. LOD scores for a genomewide scan for lifespan QTL in the DSPR. The “Chr” column indicates the chromosome arm tested (X, 2L, 2R, 3L, 3R), and the “PhysicalPosition” and “GeneticPosition” columns give information on the chromosomal position tested. Physical positions are provided as both Release 5 (“_R5”) and Release 6 (“_R6”) of the *Drosophila melanogaster* reference genome. R6 coordinates were generated from the R5 coordinates output by the DSPRqtl mapping software using the FlyBase coordinates converter tool. The “LODscore” column provides evidence for the presence of a QTL at each position along the genome. (TXT 560 kb) Additional file 5: Table S3. Protein-coding genes residing within mapped QTL intervals. The “QTL” column refers to the mapped QTL, “Cytological” and “Position_R6” provide the position of the gene in release 6 of the *D. melanogaster* reference, and “GeneSymbol” and “FBgn” provides details of the gene name. (TXT 20 kb) Additional file 6: FlyBase controlled vocabulary searches. (PDF 29 kb) Additional file 7: Table S4. Aging candidate genes extracted from FlyBase. The “FBgn”, “CG”, “GeneName”, and “Symbol” columns provide details on the gene ID. The “CytologicalLocation”, “Chr”, “PosMin_R6”, and “PosMax_R6” provide the position of the gene (R6 refers to release 6 of the *D. melanogaster* reference annotation), and “Strand” provides the orientation of the gene in the genome. The final “VocabCode” column includes a multiple digit code indicating which controlled vocabulary (CV) search term(s) the gene is associated with (see Additional file 6 for the codes); For instance, “179” in this column means the gene is associated with the CV terms “aging”, “premature aging”, and “short lived”. (TXT 44 kb) Additional file 8: Figure S2. Lifespan QTL mapped in previous studies. (PDF 52 kb) Additional file 9: Table S5. Differentially expressed genes in bodies. Table includes all genes surviving a genomewide FDR threshold of 5 % in at least one contrast. The data is taken directly from the “gene_exp.diff” Cuffdiff output file, and simply trimmed to remove data for those genes failing to reach genomewide significance (*q* < 0.05) in at least one contrast. The “Gene” and “FBgn” columns give information on the gene name, and the “Position” column provides the gene location in release 5 of the *D. melanogaster* reference genome. The “Sample1” and “Sample2” columns give the names of the two samples being compared, and the “FPKM1” and “FPKM2” columns give the FPKM (Fragments Per Kilobase of exon model per Million mapped fragments) values for each sample. The “FoldChange.Log2” column gives the log2 fold change in expression (Sample2 divided by Sample1). The “TestStat” column gives the test statistic used by Cuffdiff to compute the significance of the observed change in FPKM between samples, the “Pval” column provides the uncorrected *p*-value of the test statistic, and the “Qval” column provides the Benjamini-Hochberg FDR-adjusted *p*-value of the test statistic. (TXT 40 kb) Additional file 10: Table S6. Differentially expressed genes in heads. Table includes all genes surviving a genomewide FDR threshold of 5 %. Refer to the legend for Additional file 9: Table S5 for details of the columns. (TXT 197 kb) Additional file 11: Gene Ontology (GO) analysis summary. (PDF 45 kb) Additional file 12: Extracting genes showing age-related changes in expression from previous studies. (PDF 32 kb) Additional file 13: Figure S3. Overlap among expression candidates. (PDF 40 kb) Additional file 14: Physical positions of all differentially-expressed genes. (PDF 62 kb) Additional file 15: Table S7. RNAseq candidates within mapped QTL. The “QTL” column refers to the five QTL mapped in this study (see Table 1), and “FBgn”, “GeneSymbol”, “Cytological”, “Position_R6”, all give information on the gene. The “RNAseq_Expt” column states which study the gene was found to be differentially expressed in, “Contrast” gives the statistical test in which the gene was identified, and “ExpressionResult” gives the direction of the expression change. The “AgingCandidate” column states whether the gene is a known aging gene (see Table 2), and the “RNAseqOverlap” column gives the number of array-based expression studies identifying the gene as showing an age-related change in gene expression. (TXT 5 kb)
