# Supplementary material for: Genetic analysis of variation in lifespan using a multiparental advanced intercross Drosophila mapping population
Source: BMC Genet. 2016 Aug 2;17:113. doi: 10.1186/s12863-016-0419-9 (PMC4970266; doi:10.1186/s12863-016-0419-9)

Additional file 2: Figure S1. Block-to-block variation in lifespan.

We assayed 805 DSPR RILs for median female lifespan across four experimental blocks. There is significant variation across blocks in the average lifespan of the set of RILs assayed. The mean (± 1-SD) lifespan for each is 56.5 ± 9.93 (Block 1, 208 RILs), 50.5 ± 9.35 (Block 2, 150 RILs), 58.5 ± 10.42 (Block 3, 233 RILs), and 53.7 ± 11.98 (Block 4, 214 RILs). The figure was generated via the R "boxplot" command with standard settings.

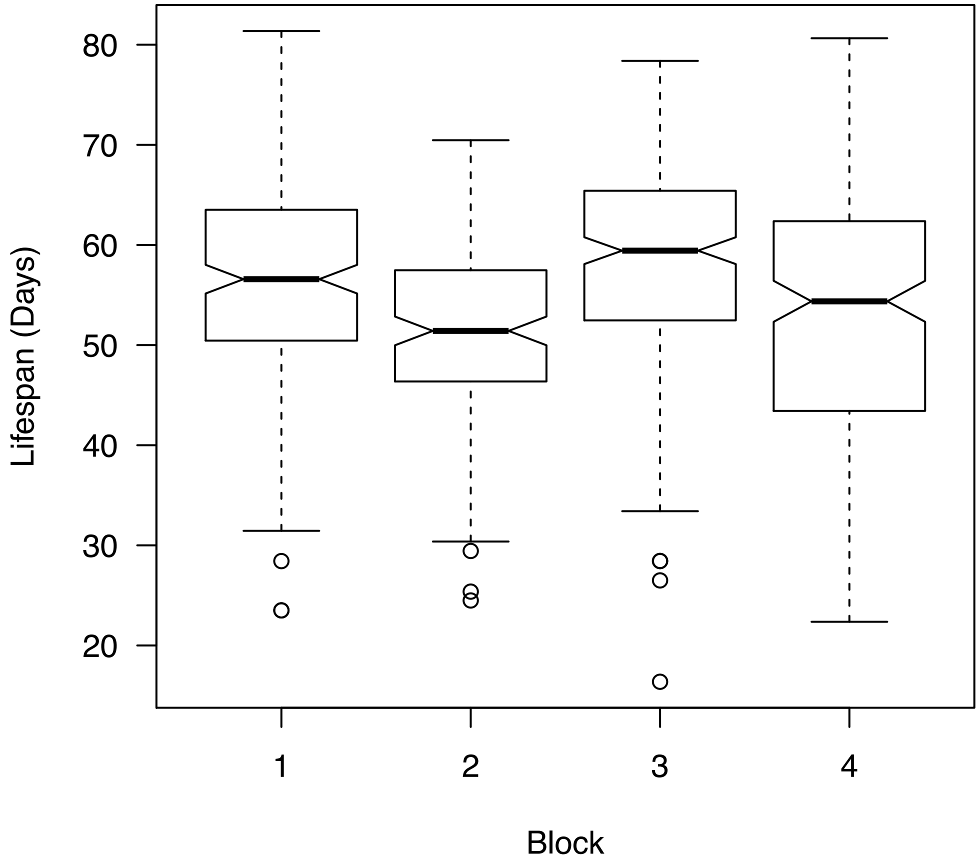

Supplement: Additional file 2: Figure S1. — Block-to-block variation in lifespan. (PDF 43 kb) [file 12863_2016_419_MOESM2_ESM.pdf]
