# Supplementary material for: Genetic analysis of variation in lifespan using a multiparental advanced intercross Drosophila mapping population
Source: BMC Genet. 2016 Aug 2;17:113. doi: 10.1186/s12863-016-0419-9 (PMC4970266; doi:10.1186/s12863-016-0419-9)
Supplement: Additional file 3: — RILs selected for RNAseq analysis. (PDF 28 kb) [file 12863_2016_419_MOESM3_ESM.pdf]

Additional file 3. RILs selected for RNAseq analysis.

The following 20 short-lived and long-lived RILs were selected for pooled RNAseq analysis of gene expression in bodies (thorax + abdomen) of females.

Short-lived body pool:

RIL	Median Lifespan (Hrs)	Median Lifespan (Days)
21086	826.2	34.4
21105	564.1	23.5
21135	780.3	32.5
21148	926	38.6
21188	754.8	31.4
21190	801.2	33.4
21220	928.2	38.7
21241	897.6	37.4
21247	850.8	35.4
21257	1067.6	44.5

Long-lived body pool:

RIL	Median Lifespan (Hrs)	Median Lifespan (Days)
21001	1783.4	74.3
21004	1663.6	69.3
21076	1807.3	75.3
21079	1831.3	76.3
21091	1639.6	68.3
21132	1663.6	69.3
21142	1689.1	70.4
21143	1713	71.4
21168	1713	71.4
21214	1639.6	68.3

The following six RILs were used for strain-by-strain analysis of gene expression in female heads.

RIL	Median Lifespan (Hrs)	Median Lifespan (Days)
21004	1663.6	69.3
21157	972.1	40.5
21168	1713.0	71.4
21210	1713.0	71.4
21214	1639.6	68.3
21250	1043.8	43.5
