# Supplementary material for: Genetic analysis of variation in lifespan using a multiparental advanced intercross Drosophila mapping population
Source: BMC Genet. 2016 Aug 2;17:113. doi: 10.1186/s12863-016-0419-9 (PMC4970266; doi:10.1186/s12863-016-0419-9)
Supplement: Additional file 6: — FlyBase controlled vocabulary searches. (PDF 29 kb) [file 12863_2016_419_MOESM6_ESM.pdf]

Additional file 6. FlyBase controlled vocabulary searches.

We searched the FlyBase controlled vocabulary (CV) terms (http://flybase.org/static_pages/termlink/termlink.html, accessed 6 January 2016) for the words "aging", "lifespan", "lived", and "longevity", and identified 9 terms, each associated with multiple genes. The number of independent genes across all terms is 568. The "CODE" associated with each term can be used along with Additional file 7: Table S4 to determine which candidate gene is associated with each term.

CODE: 1
CV ID: GO:0007568
CV term: aging
genes: 175

CODE: 2
CV ID: FBcv:0000384
CV term: aging defective
genes: 30

CODE: 3
CV ID: GO:0007569
CV term: cell aging
genes: 2

CODE: 4
CV ID: FBcv:0000791
CV term: delayed aging
genes: 2

CODE: 5
CV ID: GO:0008340
CV term: determination of adult lifespan
genes: 168

CODE: 6
CV ID: GO:0010259
CV term: multicellular organismal aging
genes: 171

CODE: 7
CV ID: FBcv:0000792
CV term: premature aging
genes: 4

CODE: 8
CV ID: FBcv:0000386
CV term: long lived
genes: 264

CODE: 9
CV ID: FBcv:0000385
CV term: short lived
genes: 503
