# Supplementary material for: Genetic analysis of variation in lifespan using a multiparental advanced intercross Drosophila mapping population
Source: BMC Genet. 2016 Aug 2;17:113. doi: 10.1186/s12863-016-0419-9 (PMC4970266; doi:10.1186/s12863-016-0419-9)

Additional file 8: Figure S2. Lifespan QTL mapped in previous studies.

We extracted the positions of QTL mapped for various lifespan/aging traits from 13 publications. All studies reported the positions of QTL intervals as cytological band locations, and the figure shows a horizontal black bar for each region implicated in each study. Alternating gray and white panels refer to the *D. melanogaster* chromosome arms (X, 2L, 2R, 3L, and 3R). The positions of the five QTL we map in the DSPR are shown as vertical red bars. Studies listed along the y-axis are: (1) Nuzhdin et al., 1997 (PMID: 9275193), (2) Leips and Mackay, 2000 (PMID: 10924473), (3) Vieira et al., 2000 (PMID: 10628982), (4) Curtsinger and Khazaeli, 2002 (PMID: 11718803), (5) Leips and Mackay, 2002 (PMID: 12227919), (6) Reiwitch and Nuzhdin, 2002 (PMID: 12688661), (7) Geiger-Thornsberry and Mackay, 2004 (PMID: 15013662), (8) Forbes et al., 2004 (PMID: 15454544), (9) Wang et al., 2004 (PMID: 15153181), (10) Nuzhdin et al., 2005 (PMID: 15834144), (11) Wilson et al., 2006 (PMID: 16702433), (12) Lai et al., 2007 (PMID: 17873888), and (13) Defays et al., 2011 (PMID: 21798333). The positions of the QTL we map overlap with those mapped in studies 6 and 13 (Q1), 3, 6, and 13 (Q2), 13 (Q3), 2, 3, 8, 11, and 13 (Q4), and 5, 6, and 10 (Q5).

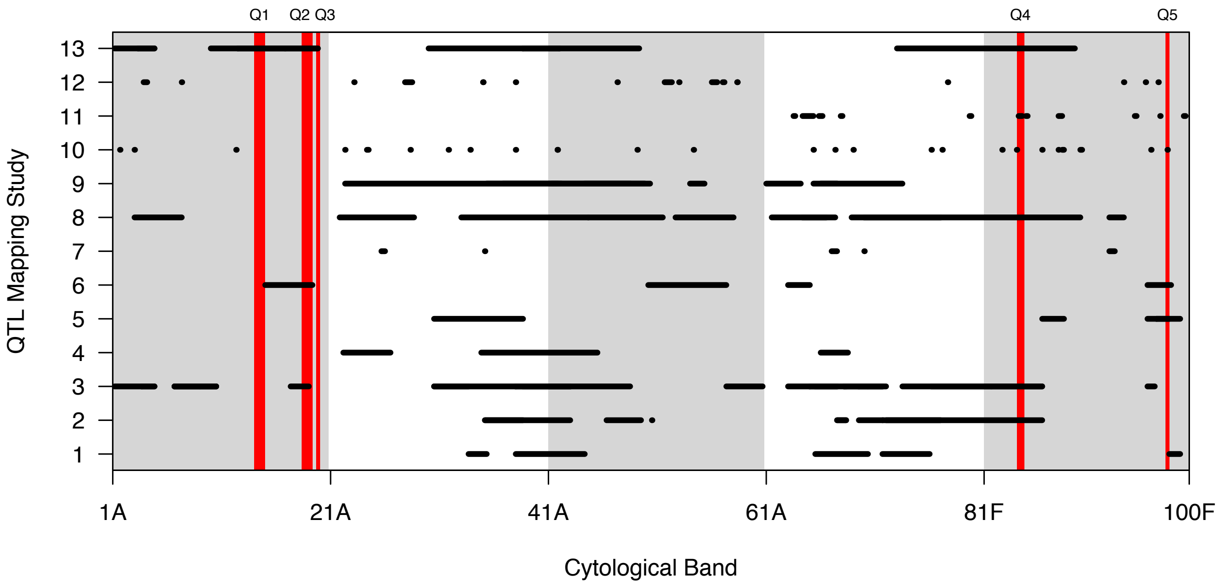

Supplement: Additional file 8: Figure S2. — Lifespan QTL mapped in previous studies. (PDF 52 kb) [file 12863_2016_419_MOESM8_ESM.pdf]
