# Supplementary material for: Genetic analysis of variation in lifespan using a multiparental advanced intercross Drosophila mapping population
Source: BMC Genet. 2016 Aug 2;17:113. doi: 10.1186/s12863-016-0419-9 (PMC4970266; doi:10.1186/s12863-016-0419-9)
Supplement: Additional file 11: — Gene Ontology (GO) analysis summary. (PDF 45 kb) [file 12863_2016_419_MOESM11_ESM.pdf]

Additional file 11. Gene Ontology (GO) analysis summary.

Gene ontology analysis was conducted via the geneontology.org site on January 27, 2016 (Analysis Type: PANTHER Overrepresentation Test - release 20150430; Annotation Version and Release Date: GO Ontology database - Released 2015-08-06). The database contained 13,690 *D. melanogaster* genes, and 245/252 (1,898/1,940) of the genes showing differential expression in bodies (heads) were present. Selected "biological process" GO category results are shown below. The "Genes" column is the total number of genes of that category in the database, "DE genes" is the number of those genes identified as differentially-expressed in our dataset, "FE" is the fold enrichment (the number of genes identified relative to the number expected by chance), and "*P*-value" is the result of a Bonferroni-corrected significance test for enrichment.

Bodies

**GO Category**	**Genes**	**DE Genes**	**FE**	***P*-value**
antibacterial humoral response (GO:0019731)<br>*All 10 increase expression with age*	29	10	>5	5.49E-07
defense response to Gram-positive bacterium (GO:0050830)<br>*All 11 increase expression with age*	41	11	>5	9.27E-07
defense response (GO:0006952)<br>*All 28 increase expression with age*	330	28	4.74	4.25E-08
egg coat formation (GO:0035803)<br>*All 5 decrease expression with age*	14	5	>5	1.71E-02
skeletal myofibril assembly (GO:0014866)<br>*All 5 decrease expression with age*	7	5	>5	5.93E-04
myofibril assembly (GO:0030239)<br>*All 10 decrease expression with age*	40	10	>5	1.15E-05

Heads

**GO Category**	**Genes**	**DE Genes**	**FE**	***P*-value**
antibacterial humoral response (GO:0019731)<br>*16/17 increase expression with age*	29	17	4.23	3.09E-03
defense response to Gram-positive bacterium (GO:0050830)<br>*All 24 increase expression with age*	41	24	4.22	2.24E-05
defense response (GO:0006952)<br>*70/84 increase expression with age*	330	84	1.84	4.63E-04
electron transport chain (GO:0022900)<br>*39/42 decrease expression with age*	86	42	3.52	2.21E-08
ATP metabolic process (GO:0046034)<br>*52/57 decrease expression with age*	133	57	3.09	8.93E-10
