# Supplementary material for: Genetic analysis of variation in lifespan using a multiparental advanced intercross Drosophila mapping population
Source: BMC Genet. 2016 Aug 2;17:113. doi: 10.1186/s12863-016-0419-9 (PMC4970266; doi:10.1186/s12863-016-0419-9)
Supplement: Additional file 12: — Extracting genes showing age-related changes in expression from previous studies. (PDF 32 kb) [file 12863_2016_419_MOESM12_ESM.pdf]

Additional file 12: Extracting genes showing age-related changes in expression from previous studies.

Pletcher et al. (2002) - PMID: 12007414
1,312 genes with age-related changes in expression.

We received information on the probe sets that showed age-related changes in gene expression from the corresponding author of this paper. The results were from an updated analysis, and a slightly larger set of significant genes were identified than was reported in the original paper. Using the annotation file provided to accompany the expression array used in the study we converted probe sets to FlyBase gene IDs ("FBgn"), ignoring any probe sets lacking FBgn. FBgn were then updated to current FBgn using FlyBase (http://flybase.org/static_pages/downloads/IDConv.html, accessed 30 December 2015). Subsequently, we retained only those unique genes with a one-to-one relationship between current and former FBgn (differences among annotation releases cause a small minority of putative open reading frames to be merged or split as annotations improve).

Landis et al. (2004) - PMID: 15136717
854 genes with age-related changes in expression.

We extracted FBgn for probe sets showing increased or decreased expression with age from Table 1 (tabs "old up", "O2 OLd UP", "old down", and "old o2 down"), and used a similar protocol as described above to filter FBgn.

Lai et al. (2007) - PMID: 17196240
2,118 genes with age-related changes in expression.

We extracted FBgn for probe sets showing increased or decreased expression with age from Supplementary Table 3. Probe sets reported to be associated with more than one gene symbol and/or FBgn were ignored. Subsequently we used the same protocol as described above to update and filter FBgn.

Zhan et al. (2007) - PMID: 17623811
3,181 genes with age-related changes in expression.

We extracted FBgn for genes with age-related expression changes from Supplementary Tables 1-7, ignoring any gene lacking an FBgn number. After updating FBgn numbers via FlyBase, we merged data from all tissues. Thus, the set of genes we employ show a significant age-related change in at least one, and perhaps as many as seven tissues.

Carlson et al. (2015) - PMID: 26090231
1,527 genes with age-related changes in expression.

We extracted FBgn for genes showing changing expression with age from Supplementary Table 1, and used the same protocol as described above to filter FBgn.
