# Supplementary material for: Genetic analysis of variation in lifespan using a multiparental advanced intercross Drosophila mapping population
Source: BMC Genet. 2016 Aug 2;17:113. doi: 10.1186/s12863-016-0419-9 (PMC4970266; doi:10.1186/s12863-016-0419-9)

Additional file 13: Figure S3. Overlap among expression candidates.

We identified 252 genes showing differential expression in body tissue between young and old animals and/or between short- and long-lived genotypes. We additionally identified 1,940 genes showing differential expression between young and old animals in heads. Comparing these lists to similar lists of age-related genes from five other publications (see Additional file 12) we found that the majority of our expression candidates had been previously identified in one or more studies. The figure shows the number of genes we identified that were unique to our study (e.g., 43 body genes), or that were found in other studies (e.g., 11 body genes were identified in all five other studies we examined).

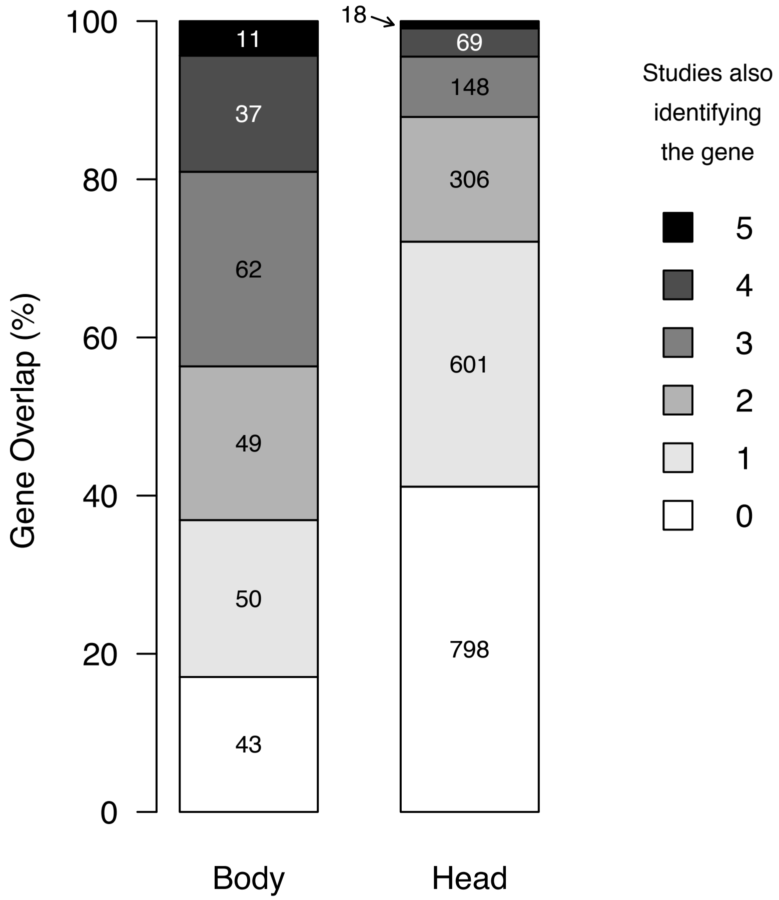

Supplement: Additional file 13: — Figure S3. Overlap among expression candidates. (PDF 40 kb) [file 12863_2016_419_MOESM13_ESM.pdf]
