# Supplementary material for: Genetic analysis of variation in lifespan using a multiparental advanced intercross Drosophila mapping population
Source: BMC Genet. 2016 Aug 2;17:113. doi: 10.1186/s12863-016-0419-9 (PMC4970266; doi:10.1186/s12863-016-0419-9)

Additional file 14. Physical positions of all differentially-expressed genes.

We plot the number of differentially-expressed (DE) genes in each 1Mb interval across the genome, separately for the two RNAseq datasets. We identified 252 differentially-expressed genes in the body (×), and 1940 in head tissue (●). Colored curves were obtained via kernel smoothing for each chromosome and dataset using the "ksmooth" function in R. The vertical gray bars represent the physical intervals of the 5 QTL mapped in the study.

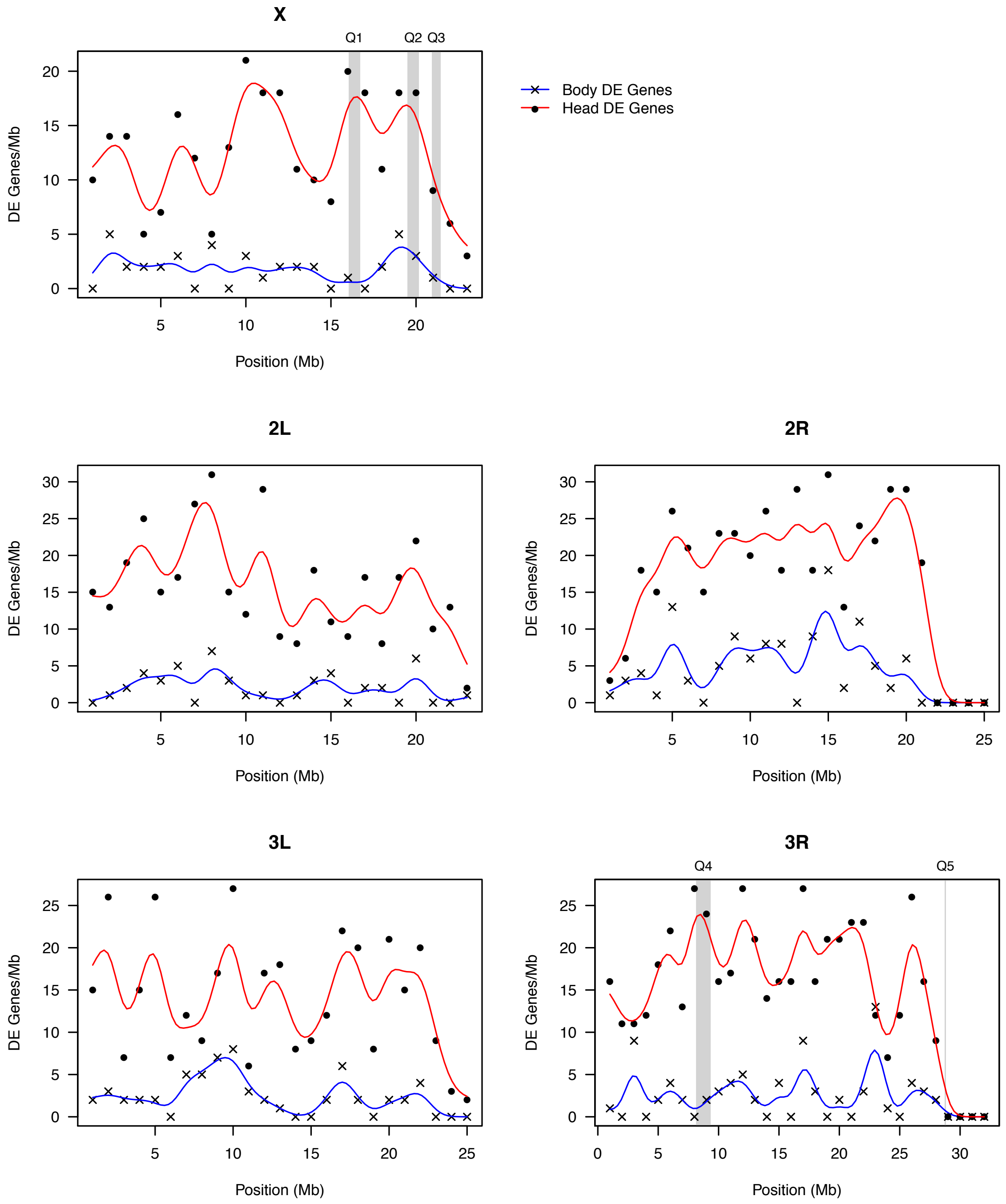

Supplement: Additional file 14: — Physical positions of all differentially-expressed genes. (PDF 62 kb) [file 12863_2016_419_MOESM14_ESM.pdf]
